# Abstract of the Investigations Thus Far Made in the Use of Equine Scarlatinal Virus

**Published:** 1884-04

**Authors:** Joseph W. Stickler

**Affiliations:** Orange, N. J.


### Art. X.—ABSTRACT OF THE INVESTIGATIONS THUS FAR MADE IN THE USE OF EQUINE SCARLATINAL VIRUS.

BY JOSEPH W. STICKLER, M. D., ORANGE, N. J.

WITH nasal mucus taken from a horse having at the time quite a severe attack of scarlatina, I inoculated twelve persons.

In each instance there were produced at, and near the point of puncture, the following changes:

Within twenty-four hours of time of inoculation there appeared considerable redness, more or less punctate. Some tenderness on pressure.

White tracings evident on second and third days.

Desquamation commencing after disappearance of redness, and continuing till the surface which had been reddened, became uncovered of epidermis.

After complete desquamation, I injected under the skin of each of the patients a few drops of human scarlatinal blood, taken from a patient who had well developed scarlatina anginosa. In no case was there any evidence of infection with the human poison.

In order that the proper value may be attached to these experiments, I think it necessary to state, that human blood taken from a person affected with scarlet fever, does not necessarily produce the disease when injected into the tissues of the lower animals. I satisfied myself on this point by inoculating a rabbit with some of the blood taken from a human scarlatinal patient. In this case there was a systemic disturbance, but it lacked the characteristic features of scarlet-fever.

On the other hand human scarlatinal blood has caused the most violent symptoms, even death. It therefore becomes necessary to test the infective power of a given sample of human scarlatinal poison upon the person to be protected, or upon others who have not been protected, in order to be absolutely certain that the disease would or would not have been produced, irrespective of the influence of equine virus. I lay special stress upon this point, because I deem it an essential one in the consideration of the subject. It is nevertheless true that some of the patients inoculated as already described, have been exposed to the poison of scarlatina as it has been conveyed to them in the atmosphere, without contracting the disease.

Since that time I have inoculated a second set of patients, all of whom had been, and were at time of inoculation, exposed to the scarlatinal contagium. The first patient was a girl of about four years of age. She had been in the room with her sister, who had scarlet-fever, since its outbreak, i. e., about six days. After inoculation with the equine virus, there was no development of the disease, although she was in constant companionship with the affected one. The next four children had been exposed several days to scarlet-fever. They were all inoculated at the same time and all developed the disease (scarlet-fever) within forty eight hours.

Two brothers were next inoculated after exposure, but neither contracted the disease. The next four children were inoculated after several day's exposure. They all contracted the diseese (scarlatina) in a mild form.

The last two cases were sisters who had been exposed several days. They were inoculated at the same time. The younger child contracted the disease within five hours after inoculation, the older, five days subsequently.

In all these cases I have estimated the duration of exposure, including the pre-eruptive stage, from eight to eleven days!

As will be observed there was abundant opportunity for impregnation of the system with the human scarlatinal poison prior to the use of the equine virus. In this manner may be explained, possibly, the failures above recorded. In every case in which the disease developed after the second day from time of inoculation, the symptoms were very mild.

I have also inoculated two colts, and two calves with human scarlatinal virus. The results are as follows:

First colt, about one year old. General condition good. No discharge from nostrils. Mucous membranes of eyelids, nose and mouth, normal in appearance. Temperature before inoculatlon $101\frac{1}{4}°$ F. After throwing the colt I injected some of the blood serum (scarlatinal) into the right jugular vein. I also introduced under the skin of the thorax a blood-clot from the same patient, and gave per orem, some pharyngeal mucus. The temperature gradually rose till it reached 102° F. The temperature reached the highest point May 14th, on which day it was $103\frac{1}{2}°$ F. During the time the temperature was above the normal, the mucous membranes of the eyes, nostrils and pharynx were injected, and from the nostrils there was a discharge of ropy mucus. There was a slight sore throat which disappeared with the fading of the injection of the mucous membranes. There was no oedema of the sheath or extremities afterwards.

Second colt, one year old. Her temperature for four successive days before inoculation was as follows: June 18th,

$101\frac{3}{4}^\circ$ F.; June 19th, $101\frac{3}{4}^\circ$ F.; June 20th, $101\frac{3}{4}^\circ$ F.; June 21st, $101\frac{3}{4}^\circ$ F. The condition of the colt was good. June 28th, enveloped her head in a bag, containing a chemise which had been worn by a scarlet-fever patient; July 2d, injected into left jugular vein, and into vein of left leg some human scarlatinal blood; July 9th, introduced under skin of abdomen some epidermal scales. Within forty-eight hours after the first inoculation the temperature began to rise, the colt became less active and was disposed to keep quiet. Cough manifested itself on the sixth day and with it there was some difficulty in swallowing, associated with tenderness of throat on handling. The visible mucous membranes were quite red, and at certain points had the appearance of the mucous membrane of a mans pharynx when affected by scarlet-fever. The inter-maxillary glands were enlarged and sensitive. There was a discharge of tenacious mucus from the nostrils. The interior of the pharynx was unusually red. The temperature during the time the mucous membranes were red, ranged between $101\frac{3}{4}^\circ$ F., and $103^\circ$ F. There was no swelling of the extremities or symptoms of renal complications.

The first calf was inoculated without apparent result.

The second calf was exposed to the contagium of human scarlatina by having its head enveloped in a chemise worn by a patient. Chemise applied October 23d. November 5th, temperature was $103\frac{3}{4}^\circ$ F. There was a slight discharge of mucus from nostrils. The occular and palpebral conjunctiva were somewhat reddened. November 16th, noticed quite an intense redness of the interdigital spaces. Mucous membrane of nose *very* red. Thin mucous discharge from either nostril. Papules upon ears. Rather offensive diarrhœa.

The diarrhœa became worse, the abdomen markedly tympanitic, loss of strength, extreme and finally death supervened on November 22d. When the last calf was brought to me, it was not in a perfectly normal condition, although not suffering from a special complaint. It was weak, but had no gastro-intestinal disturbance. Its eyes and nostrils were also in good condition. Its skin was somewhat scurfy. I have endeavored to give simple facts in recording these results, and as I have watched the changes wrought in the lower animals, especially colts, by the introduction into their tissues of human scarlatinal poison I have been encouraged to believe that a reliable virus can be obtained which will have the power to protect the human system against scarlatina, which is one of the worst diseases to which man is liable.

